# Home Parenting Environment and Executive Function Development in 5- to 6-Year-Old Children: A Three-Wave Longitudinal Study Using Multivariate Latent Growth Modeling

**DOI:** 10.3390/bs16081415

**Published:** 2026-08-18

**Authors:** Song Shi, Mengqi Guan, Mengyao Yu, Jiaman Wang

**Affiliations:** 1 School of Educational Science, Nantong University, Nantong 226019, China; liuchang861012@163.com (S.S.); ymy1148074962@163.com (M.Y.); 2 School of Educational Science, Kashgar University, Kashgar 844000, China; wjm020630@163.com

**Keywords:** home parenting environment, executive function, 5- to 6-year-old children, developmental trajectories, multivariate latent growth modeling

## Abstract

Executive function is important for children’s self-regulation and school readiness, and the home parenting environment is a key proximal context for its development. However, less is known about whether the overall home parenting environment is associated with children’s initial executive function and subsequent change. This study examined these associations among 265 children aged 5–6 years in Yancheng, China, using three-wave data collected at six-month intervals. At each wave, mothers rated children’s executive function using the Behavior Rating Inventory of Executive Function–Preschool Version (BRIEF-P) and reported on the home parenting environment. Baseline language ability was assessed using the Peabody Picture Vocabulary Test-Revised (PPVT-R). Multivariate latent growth modeling was conducted, controlling for child sex and baseline language ability. Executive function increased significantly over one year, with individual differences in initial level and growth rate. The home parenting environment showed no significant mean-level change, although its initial level differed across families. Higher initial levels of the home parenting environment were associated with higher initial executive function, *β* = 0.446, *p* < 0.001, and faster executive function growth, *β* = 0.409, *p* = 0.004. These findings highlight the potential role of a supportive home parenting environment in children’s executive function development.

## 1. Introduction

The ages of 5 to 6 years constitute an important period of rapid executive function development (Castellanos-Ryan et al., 2023). During this period, executive function is closely associated not only with children’s school readiness and classroom adjustment but also with their social development and subsequent internalizing and externalizing problems (Stucke & Doebel, 2024; Yang et al., 2022). Examining executive function during this period is therefore important for understanding early changes in cognitive regulation and their implications for later academic and social adjustment.

Executive function development is not simply a by-product of age-related maturation. Prior research has shown that executive function in early childhood develops in relation to language ability and is also shaped by children’s everyday developmental environments (Carolus et al., 2024; Filipe et al., 2023). Among these environments, the family represents one of the earliest, most immediate, and most enduring contexts for young children’s development (Draper et al., 2024; Jiang et al., 2024). Existing studies have examined associations between family experiences and executive function from several perspectives, including family socioeconomic status, parent–child interaction, positive parenting, autonomy support, and the home learning environment (Castelo et al., 2022; Fan et al., 2022). Taken together, this body of work suggests that the family is not merely a background condition for child development. Rather, through daily interactions, rule-setting, emotional responses, and supportive practices, family experiences may provide recurring opportunities for children to practice and strengthen executive regulation, particularly when parents offer warmth, structure, and autonomy support (Distefano et al., 2024; Rungsattatharm et al., 2025; Wang & Gai, 2024).

Existing research has largely focused on specific family factors, with comparatively less attention to the home parenting environment as a multidimensional and integrated context. Consequently, how the overall quality of the home parenting environment is associated with children’s executive function development remains insufficiently understood. At the same time, prior studies have primarily examined static associations or single-time-point differences, leaving limited evidence regarding how the home parenting environment is associated with both children’s initial level of executive function and subsequent change. To address these limitations, the present study focused on 5- to 6-year-old children and used three-wave longitudinal data collected at six-month intervals. Using multivariate latent growth modeling, the study first examined the developmental trajectories of children’s executive function and the home parenting environment and then tested whether the initial level of the home parenting environment, conceptualized as a relatively stable family background factor, was associated with children’s initial level of executive function and their growth rate over one year.

### 1.1. Conceptualization and Development of Executive Function

Executive function is commonly defined as a set of higher-order cognitive processes that enable individuals to maintain and update information, inhibit inappropriate responses, and flexibly shift attention or strategies during goal-directed activities. Its core components are generally considered to include working memory/updating, inhibitory control, and cognitive flexibility (Diamond, 2013; Pitta, 2026). For 5- to 6-year-old children, executive function is not only reflected in performance on specific cognitive tasks, but also provides a foundation for rule-following, behavioral regulation, task engagement, and school readiness (Cumming et al., 2022; Zakharova et al., 2022). Prior reviews and empirical studies have shown that stronger executive function in early childhood is associated with more adaptive learning behaviors, better classroom functioning, and more favorable social, behavioral, and emotional outcomes, including fewer internalizing, externalizing, and attention problems (Stucke & Doebel, 2024; Turnbull et al., 2024). Executive function in preschool is also closely related to school readiness and social–emotional competence, suggesting that early regulatory capacities have implications beyond cognitive performance alone (Boise & Knoche, 2024).

Recent research has increasingly moved beyond single-time-point comparisons and has begun to examine executive function as a developmental process (Castellanos-Ryan et al., 2023). Longitudinal evidence suggests that executive function undergoes substantial change across early childhood, with children differing not only in their initial levels but also in their rates of growth (Chu & Joseph, 2024). Language development is also closely related to changes in executive function during this period, as language may support children’s ability to understand rules, maintain goals, and regulate behavior (Bruce et al., 2023). These findings indicate that executive function should not be treated as a static ability measured at a single point in time. Instead, it is better understood as a developing regulatory capacity whose initial status and subsequent growth may vary across children (Reilly et al., 2022). A longitudinal approach is therefore particularly useful for capturing the developmental characteristics of executive function among 5- to 6-year-old children.

Although executive function is multidimensional, its components are interrelated during the preschool years and show substantial conceptual overlap with broader self-regulatory capacities (Heinze et al., 2025; Vanhala et al., 2023). This is especially relevant when the research aim is to examine how family experiences are associated with children’s developmental trajectories rather than to distinguish the mechanisms of separate executive function components. Treating executive function as an integrated latent construct is therefore theoretically appropriate for capturing its overall developmental pattern in early childhood (Castellanos-Ryan et al., 2023; Reilly et al., 2022). Accordingly, the present study focuses on the overall trajectory of executive function as an integrated regulatory capacity, rather than examining the differential developmental mechanisms of its sub-components.

### 1.2. Conceptualization and Relative Stability of the Home Parenting Environment

Bronfenbrenner’s bio-ecological perspective provides the overarching theoretical framework for understanding the role of the home parenting environment in this study. From this perspective, the family is one of the earliest and most enduring microsystems in which young children develop, and recurring proximal processes within this microsystem constitute a primary pathway through which development unfolds (Bronfenbrenner & Morris, 2006). Accordingly, the home parenting environment can be understood as a multidimensional developmental context shaped by everyday family interactions, emotional support, behavioral expectations, learning opportunities, and material or relational resources (Draper et al., 2024; Gregoriadis & Evangelou, 2022). It is therefore not equivalent to a single parenting practice, nor is it simply the sum of several isolated family variables. Rather, it reflects the broader quality and organization of children’s proximal experiences within the family system (Hanlin & Zhou, 2024). This conceptualization allows the present study to examine the home parenting environment as an integrated contextual condition associated with both children’s initial executive function and its subsequent developmental trajectory.

A key feature of the home parenting environment is its relative continuity across the preschool years. Although specific parenting behaviors and family routines may change, longitudinal studies suggest that parenting patterns and children’s routines often show meaningful cross-time continuity during early childhood (Cheng et al., 2025; Selman et al., 2026). Within the bio-ecological framework, such continuity is theoretically important because repeated experiences within a relatively stable microsystem may accumulate over time and become developmentally consequential. The home parenting environment may therefore function as an enduring contextual condition that structures children’s everyday opportunities for interaction, learning, and self-regulation. Children’s executive function should likewise be understood as developing through ongoing exchanges between individual characteristics and ecological conditions, with environmental risks and protective experiences potentially contributing to differences in regulatory development (Merculief et al., 2024).

### 1.3. Home Parenting Environment and Executive Function in 5- to 6-Year-Old Children

Existing evidence on family experiences and children’s executive function can be summarized across three dimensions: parent–child interaction, cognitive support, and family organization. Within parent–child interactions, caregiver sensitivity, responsiveness, and appropriate support may provide children with opportunities to practice attention control, behavioral inhibition, and emotion regulation. Emotional and cognitively supportive parenting has generally been associated with stronger executive function, whereas intrusive interaction has been linked to poorer outcomes (Koşkulu-Sancar et al., 2023). Sensitive caregiving and parental responsiveness have also been related to later executive function (Andrews et al., 2021; Werchan et al., 2023). Cognitive support, including language-rich interaction, shared reading, and stimulating activities, may help children understand rules, maintain goals, and organize behavior. For example, maternal language input during shared reading has been associated with subsequent language and executive function development (Venkadasalam et al., 2022). Family organization also matters: household chaos may disrupt routines and behavioral guidance and has been associated with poorer executive function, partly through reduced parental responsiveness (Andrews et al., 2021). Similar associations have been reported among young children in China (Zhan & Guo, 2023), although longitudinal findings are not fully consistent. Fung et al. (2023), for instance, found that economic pressure predicted later working memory and attentional shifting, whereas household chaos did not significantly predict specific executive-function components. Overall, supportive interaction, cognitive stimulation, and family organization jointly form the broader home context associated with children’s executive function.

Despite growing evidence linking these aspects of family experience to children’s executive function, two issues remain insufficiently addressed. Previous studies have generally examined family socioeconomic status, cognitive stimulation, maternal sensitivity, and household chaos as separate predictors of executive function (Koşkulu-Sancar et al., 2023; Lohndorf et al., 2021). Although this approach has yielded valuable evidence regarding the role of individual family characteristics, it may reduce children’s family experiences to a set of isolated indicators and therefore fail to capture the broader role of the home parenting environment as a multidimensional and integrated developmental context. Emotional support, cognitive stimulation, behavioral guidance, involvement in children’s activities, and the organization of family life do not operate independently; rather, they jointly constitute the family environment that children experience in everyday life. A more holistic perspective is therefore needed to clarify how the overall quality of the home parenting environment is associated with children’s executive function. Although some longitudinal studies have examined combinations of early family characteristics, they have focused on risk profiles, income, learning materials, or specific parenting factors rather than the multidimensional home parenting environment (Ku & Blair, 2023; Murphy et al., 2022). Evidence therefore remains limited regarding whether relatively stable differences in the overall home parenting environment are associated not only with children’s initial level of executive function but also with their subsequent rate of growth. This issue is particularly salient for 5- to 6-year-old children, who are approaching the transition from preschool to primary school and facing increasing demands for rule-following, attentional control, task persistence, and behavioral regulation. Accordingly, conceptualizing the home parenting environment as an integrated and relatively stable family context may provide a clearer understanding of individual differences in both the initial level and developmental pace of executive function during this period.

### 1.4. Child-Level Covariates: Sex and Language Ability

When examining associations between the home parenting environment and children’s executive function trajectories, it is necessary to account for basic child-level differences that may be related to executive function ratings. Sex is commonly included as a covariate in studies of executive function development. However, evidence on sex differences in early executive function remains mixed. Such differences may depend on task demands, measurement approaches, and informant sources. Although girls may show a slight advantage on some simple inhibitory control tasks, the effect appears small and does not necessarily indicate a stable sex advantage in overall executive function (Grissom & Reyes, 2019; Silverman, 2021).

Language ability is another important child-level factor to consider. Better receptive vocabulary and language comprehension may help children understand rules, hold task goals in mind, regulate attention, and inhibit inappropriate responses. Longitudinal evidence also suggests that language ability and executive function are closely related during the preschool years and may develop in mutually reinforcing ways (Bruce et al., 2023; Xing et al., 2022). Therefore, the present study included child sex and baseline language ability as covariates to reduce potential confounding from individual differences and to more clearly examine whether the home parenting environment was associated with children’s initial executive function and subsequent growth.

### 1.5. Developmental Significance of Ages 5 to 6 Years

The ages of 5 to 6 years are a particularly meaningful period for studying executive function development. Most children at this age are approaching the transition from preschool to primary school, a period in which executive function continues to develop and becomes increasingly relevant to school readiness, classroom adjustment, and later academic performance (Memba & Ostrov, 2025; Reilly et al., 2022). As children move toward more structured school contexts, they are expected to sustain attention, follow rules, persist in tasks, inhibit impulsive responses, and flexibly adjust their behavior. These demands make executive function especially important for children’s early adaptation to school settings (Castellanos-Ryan et al., 2023; Turnbull et al., 2024). Self-regulation is a key developmental capacity in early childhood because it supports children’s attention, rule-following, emotion regulation, impulse control, and school readiness (Ernst & Stelley, 2024).

This developmental period is also important because children’s self-regulatory growth remains closely embedded in everyday family experiences. Family interactions, rule-setting, emotional support, and activity organization may provide repeated opportunities for children to practice attention control, behavioral regulation, planning, and flexible adjustment (Hanlin & Zhou, 2024; Ku & Blair, 2023). At ages 5 to 6, children are increasingly able to understand rules, engage in goal-directed activities, and respond to adult guidance, making this a particularly informative period for examining how the home parenting environment relates to executive function development (Rungsattatharm et al., 2025). As an integrated context involving emotional support, behavioral expectations, cognitive stimulation, and daily interaction, the home parenting environment may provide a continuing experiential basis for executive function development (Koşkulu-Sancar et al., 2023). Focusing on this age group therefore helps clarify how differences in the family context are linked to both the starting point and developmental pace of executive function during the preschool-to-primary school transition.

### 1.6. The Present Study

Although prior research has linked family experiences to children’s executive function through factors such as family socioeconomic status, positive parenting, and the home learning environment, two issues require further investigation. First, previous studies have often represented the family context using separate indicators, with comparatively less attention to the home parenting environment as a multidimensional and integrated developmental context. Consequently, it remains unclear how the overall quality of children’s everyday family experiences is associated with executive function development. Second, much of the existing evidence has been based on cross-sectional differences or associations between selected family factors and executive function assessed at a single time point. Less is known about whether relatively stable differences in the home parenting environment are associated not only with children’s initial level of executive function but also with their subsequent rate of growth.

To address these issues, the present study focused on 5- to 6-year-old children, who are approaching the transition from preschool to primary school. Using three-wave longitudinal data collected at six-month intervals, the study applied multivariate latent growth modeling to examine the developmental trajectories of children’s executive function and the home parenting environment. After establishing these trajectories, we tested whether the initial level of the home parenting environment was associated with both the initial level and growth rate of executive function over one year, while controlling for child sex and baseline language ability. By incorporating the home parenting environment as an integrated family context into a developmental trajectory model, the present study sought to provide a more nuanced understanding of the family environmental basis of executive function development during this transitional period. The findings may also provide an empirical basis for identifying potentially modifiable features of the home parenting environment and inform the design and evaluation of future family-based interventions aimed at supporting executive function development during the transition from preschool to primary school.

Based on the above rationale, three hypotheses were proposed:

**Hypothesis 1.**  *Across the three waves, children’s executive function would show significant mean-level change, with significant individual differences in both initial level and rate of change.*

**Hypothesis 2.**  *The initial level of the home parenting environment would be positively associated with children’s initial level of executive function.*

**Hypothesis 3.**  *The initial level of the home parenting environment would be positively associated with children’s growth rate of executive function over one year.*

Child sex and baseline language ability were included as covariates to test whether these associations remained robust after accounting for basic child-level differences.

## 2. Materials and Methods

### 2.1. Participants and Procedure

This study employed a three-wave longitudinal design. The target population consisted of children aged approximately 5 years who were enrolled in middle kindergarten classes in Yancheng, Jiangsu Province, China. The sampling frame comprised the enrollment rosters of children who met these criteria within the study area, and participants were selected at the individual child level using a random number table. A total of 322 children were recruited as the initial sample. Follow-up assessments were conducted at six-month intervals, resulting in a one-year study period by the completion of the third wave.

At each wave, mothers reported on children’s executive function and the home parenting environment. At baseline, demographic information was collected, and children’s language ability was individually assessed by trained researchers. The study was approved by the Ethics Review Committee of the School of Educational Science, Nantong University (approval no. NT202406; 19 June 2024). Before data collection, written informed consent was obtained from a parent or legal guardian of each participating child. Families were informed that participation was voluntary and that they could withdraw at any time.

Attrition occurred across the three waves because of school transfer, sick leave, or absence on the day of assessment. Twenty-one children were lost from Time 1 to Time 2, and an additional 36 children were lost from Time 2 to Time 3. The final analytic sample included 265 children who completed all three waves, including 146 boys (55.1%) and 119 girls (44.9%). Children who did not complete all three waves were included only in attrition analyses and were not retained in the subsequent latent growth models. Attrition analyses indicated no significant differences between retained and attrited participants in child sex, baseline language ability, Time 1 home parenting environment, or Time 1 executive function, with all *p* values greater than 0.05. These results suggest that attrition did not show clear evidence of systematic bias.

### 2.2. Measures

#### 2.2.1. Executive Function

Children’s executive function was measured using the Behavior Rating Inventory of Executive Function–Preschool Version (BRIEF-P; Gioia et al., 2003), based on the validated Chinese parent-report version (Lu et al., 2017). The BRIEF-P contains 63 items and assesses five domains of preschool executive function: Inhibit, Shift, Emotional Control, Working Memory, and Plan/Organize. The Chinese parent-report version has demonstrated satisfactory reliability and construct validity. Confirmatory factor analysis supported its first-order five-factor structure, *χ*^2^(1885) = 3075.77, *χ*^2^/df = 1.63, NNFI = 0.91, CFI = 0.92, and RMSEA = 0.05. Test–retest reliability coefficients ranged from 0.54 to 0.72, and internal consistency coefficients ranged from 0.78 to 0.95 (Lu et al., 2017). In the present study, mothers rated each item based on the child’s everyday behavior using a 3-point scale ranging from 1 (never) to 3 (often). The BRIEF-P primarily assesses difficulties in executive function and self-regulation. Therefore, all items were reverse-coded before the mean score was calculated, such that higher scores indicated better executive function. The scale demonstrated good internal consistency across the three waves, with Cronbach’s α values of 0.94, 0.96, and 0.96 at T1, T2, and T3, respectively.

#### 2.2.2. Home Parenting Environment

The home parenting environment was measured using the Home Parenting Environment Questionnaire for 3- to 6-Year-Old Children, a Chinese measure developed to assess the overall quality of young children’s everyday family environment (He, 2008). The questionnaire contains 53 items covering six dimensions: language and cognitive stimulation, emotional warmth and self-expression, social adaptation and self-care, neglect/interference/punishment, activity diversity and play involvement, and home atmosphere and physical space. The original scale-development study provided evidence of good reliability and construct validity. Cronbach’s α was 0.93 for the overall scale and ranged from 0.70 to 0.87 across the six dimensions, while the two-month test–retest reliability coefficient for the overall scale was 0.72. Confirmatory factor analysis supported the proposed six-factor structure, *χ*^2^/df = 2.13, CFI = 0.92, RMSEA = 0.05 (He, 2008).

In the present study, mothers rated each item on a 5-point scale ranging from 1 (never) to 5 (always). Items in the neglect/interference/punishment dimension were reverse-coded, after which the mean score across all items was calculated. Higher scores indicated a higher-quality home parenting environment. The questionnaire demonstrated good internal consistency across the three waves, with Cronbach’s α values of 0.94, 0.94, and 0.95 at T1, T2, and T3, respectively.

#### 2.2.3. Baseline Language Ability

Children’s baseline language ability was assessed using the Peabody Picture Vocabulary Test-Revised (PPVT-R), following the revised norms developed for children in urban Shanghai, China (Sang & Miao, 1990). The PPVT-R is an individually administered measure of receptive vocabulary and language comprehension. It consists of 120 picture sets, with each set containing four pictures. During the assessment, a trained examiner orally presented a target word, and the child was asked to select the picture that best matched the meaning of the word. Each correct response was scored as 1, and each incorrect response was scored as 0. The basal criterion was established when the child answered eight consecutive items correctly, and the ceiling criterion was reached when the child answered six consecutive items incorrectly. The total number of correct responses was used as the language ability score, with higher scores indicating stronger receptive vocabulary and language comprehension.

### 2.3. Data Analysis

All main analyses were conducted using the complete-case analytic sample of children who participated in all three waves (N = 265). First, descriptive statistics, correlation analyses, and repeated-measures analyses of variance were conducted in IBM SPSS Statistics 27.0 (IBM Corp., Armonk, NY, USA) to examine the basic characteristics of the study variables and their mean-level changes across time. Subsequently, unconditional latent growth models were specified separately for children’s executive function and the home parenting environment in Mplus 8.3 (Muthén & Muthén, Los Angeles, CA, USA) to estimate their intercepts, linear slopes, and individual differences in initial level and rate of change. A conditional latent growth model for executive function was then estimated by including child sex and baseline language ability as covariates. Finally, a multivariate latent growth model was constructed to examine the longitudinal association between the home parenting environment and children’s executive function trajectories. In this model, child sex and baseline language ability were controlled, and the main paths of interest were from the initial level of the home parenting environment to the initial level and linear slope of children’s executive function. Based on the results of the unconditional growth model, the home parenting environment was treated as a relatively stable family background characteristic in the subsequent model, with its initial level used to represent between-family differences in the overall quality of the home parenting environment.

## 3. Results

### 3.1. Assessment of Common Method Bias

Because children’s executive function and the home parenting environment were both reported by mothers, Harman’s single-factor test was conducted separately at each wave to assess potential common method bias. At each wave, all item-level indicators of the two constructs were entered into an unrotated exploratory factor analysis. The first factor accounted for 18.25%, 21.16%, and 17.38% of the total variance at T1, T2, and T3, respectively. All three values were well below the 40% criterion adopted in this study, suggesting that common method bias was not severe.

### 3.2. Descriptive Statistics

Table 1 presents the means, standard deviations, and bivariate correlations among the study variables.

Children’s executive function increased from T1 to T3. A repeated-measures ANOVA showed a significant effect of time on executive function. Mauchly’s test indicated that the assumption of sphericity was violated, *W* = 0.94, *χ*^2^(2) = 16.82, *p* < 0.001; therefore, Greenhouse–Geisser correction was applied. The time effect remained significant after correction, *F*(1.88, 497.21) = 27.30, *p* < 0.001, *ηp*^2^ = 0.09. For the home parenting environment, no significant mean-level change was observed across the three waves. Mauchly’s test indicated that the assumption of sphericity was met, *W* = 0.997, *χ*^2^(2) = 0.86, *p* = 0.65. The within-subjects effect was not significant, *F*(2, 528) = 1.31, *p* = 0.271, *ηp*^2^ = 0.005. Correlation analyses showed that baseline language ability, measured by the PPVT-R, was positively associated with children’s executive function at all three waves. Children’s executive function was also positively associated with the home parenting environment across the three waves, suggesting that higher levels of the home parenting environment were related to better executive function performance. Child sex was not significantly associated with either executive function or the home parenting environment at any wave.

### 3.3. Unconditional Latent Growth Model for Executive Function

An unconditional latent growth model was estimated to examine the developmental trajectory of children’s executive function. Given the equal six-month intervals across the three waves, the slope factor loadings were fixed at 0, 1, and 2, respectively. Thus, the intercept represented the initial level of executive function at T1, and the slope represented linear change per six-month interval.

The unconditional linear growth model showed acceptable fit to the data (Table 2), *χ*^2^(1) = 0.53, *p* = 0.47, CFI = 1.00, TLI = 1.01, RMSEA = 0.00, 90% CI [0.00, 0.15], SRMR = 0.01. The intercept mean was 2.36, *p* < 0.001. The slope mean was positive and significant, *M* = 0.07, *p* < 0.001, indicating that children’s executive function increased significantly from T1 to T3. The intercept variance was significant, *σ*^2^ = 0.07, *p* < 0.001, suggesting significant individual differences in children’s initial level of executive function. The slope variance was also significant, *σ*^2^ = 0.01, *p* < 0.05, indicating that children differed in their rate of executive function growth. The intercept–slope association was not significant, *r* = 0.04, *p* = 0.86, suggesting that children’s initial level of executive function was not significantly related to their subsequent rate of growth.

### 3.4. Unconditional Latent Growth Model for the Home Parenting Environment

A separate unconditional linear growth model was estimated for the home parenting environment, with slope factor loadings fixed at 0, 1, and 2 to represent linear change across the three equally spaced waves.

In the freely estimated model, the slope variance for the home parenting environment yielded a small negative estimate close to zero, indicating an improper boundary estimate. Therefore, the slope variance was fixed to zero, and the model was re-estimated. The revised model fit the data well (Table 2), *χ*^2^(3) = 0.85, *p* = 0.84, CFI = 1.00, TLI = 1.00, RMSEA = 0.00, 90% CI [0.00, 0.06], SRMR = 0.04. The intercept mean was 3.86, *p* < 0.001. The slope mean was positive but not statistically significant, *M* = 0.02, *p* = 0.189, indicating that the home parenting environment did not show significant mean-level change over the one-year period. The intercept variance was significant, *σ*^2^ = 0.182, *p* < 0.001, suggesting meaningful between-family differences in the initial level of the home parenting environment. Because the slope variance was fixed to zero, there was no evidence of significant individual differences in the rate of change in the home parenting environment. On this basis, the initial level of the home parenting environment was treated in subsequent analyses as a relatively stable family background characteristic.

### 3.5. Conditional Latent Growth Model for Executive Function: Roles of Sex and Baseline Language Ability

To examine whether child-level characteristics were associated with children’s executive function trajectories, child sex and baseline language ability were added to the unconditional linear growth model for executive function.

The conditional latent growth model showed good fit to the data (Table 2), *χ*^2^(3) = 0.92, *p* = 0.82, CFI = 1.00, TLI = 1.03, RMSEA = 0.00, 90% CI [0.00, 0.06], SRMR = 0.01. Baseline language ability, as measured by the PPVT-R, was positively associated with the intercept of executive function, *β* = 0.30, *p* < 0.001, indicating that children with higher baseline language ability showed higher initial levels of executive function. Child sex, coded as 1 = boys and 2 = girls, was not significantly associated with the intercept, *β* = 0.04, *p* = 0.61. Neither child sex nor baseline language ability was significantly associated with the slope of executive function. Specifically, the association between child sex and the executive function slope was nonsignificant, *β* = 0.08, *p* = 0.38, and the association between baseline language ability and the slope was also nonsignificant, *β* = 0.13, *p* = 0.20. These results suggest that baseline language ability was related to children’s initial executive function but not to their rate of growth over the one-year period, whereas child sex was not significantly related to either the initial level or the growth rate of executive function.

### 3.6. Associations Between Executive Function Trajectories and the Initial Level of the Home Parenting Environment

A multivariate latent growth model was estimated to examine whether the initial level of the home parenting environment was associated with children’s executive function trajectories after controlling for child sex and baseline language ability. Based on the temporal ordering of the variables and the study hypotheses, the primary paths of interest were specified from the intercept of the home parenting environment to the intercept and linear slope of executive function.

The model fit was mixed, *χ*^2^(16) = 46.17, *p* < 0.001, CFI = 0.95, RMSEA = 0.08, 90% CI [0.06, 0.11], and SRMR = 0.06. The CFI and SRMR supported an acceptable level of fit, whereas the significant chi-square statistic, the TLI of 0.92, and the upper bound of the RMSEA confidence interval indicated only moderate fit. Within this model, the initial level of the home parenting environment was positively associated with the initial level of children’s executive function, *β* = 0.45, *p* < 0.001. Thus, children with a higher initial level of the home parenting environment tended to have higher executive function at T1. The initial level of the home parenting environment was also positively associated with the linear slope of executive function, *β* = 0.41, *p* = 0.004. After accounting for child sex and baseline language ability, a higher initial level of the home parenting environment was associated with a steeper positive slope of executive function over the one-year study period (Table 3).

**Figure 1:**
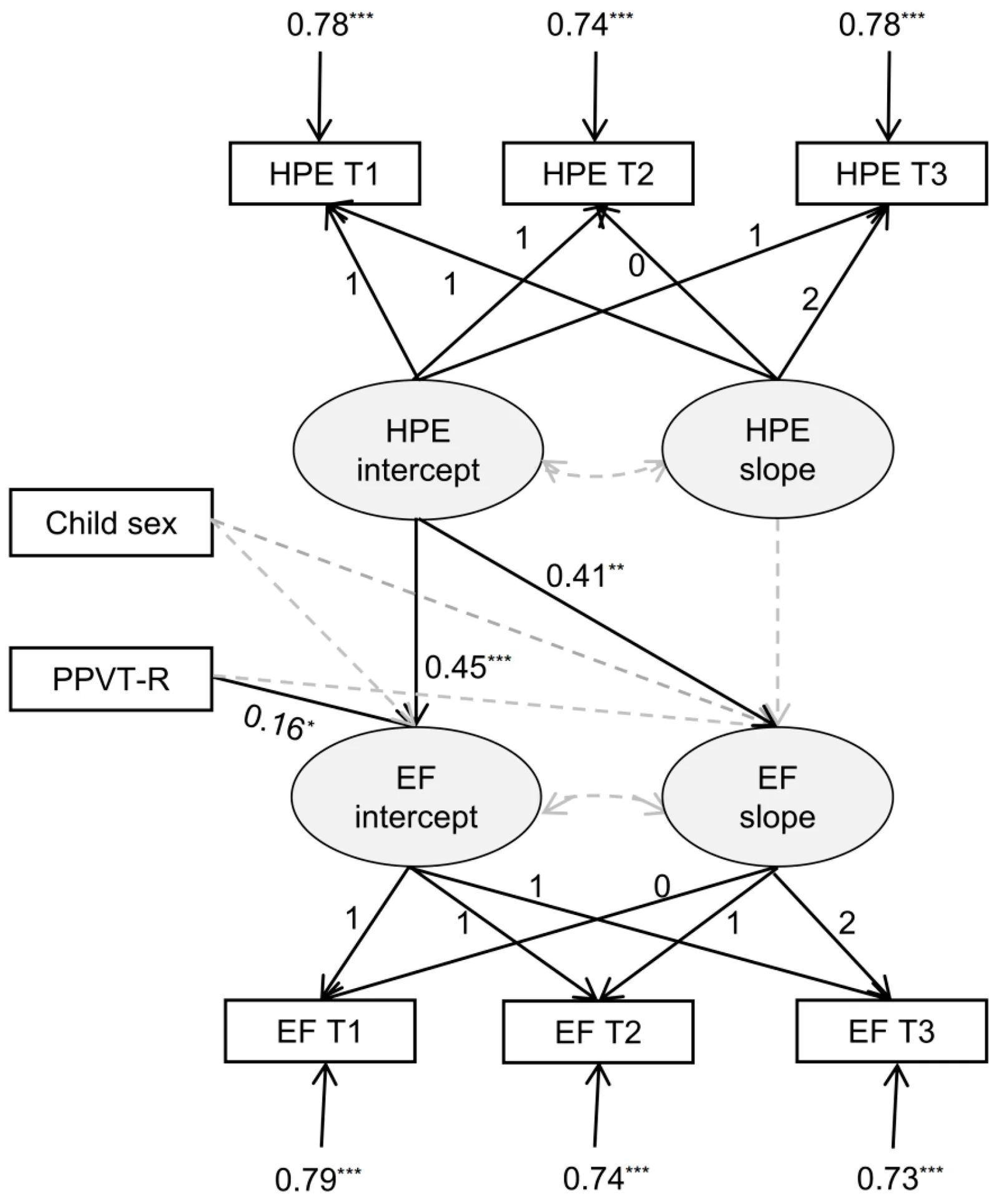
Multivariate latent growth model of the home parenting environment and executive function. Standardized coefficients are shown. Solid lines indicate statistically significant paths, and dashed lines indicate nonsignificant paths. HPE = home parenting environment; EF = executive function; PPVT-R = Peabody Picture Vocabulary Test-Revised. * *p* < 0.05. ** *p* < 0.01. *** *p* < 0.001.

The standardized coefficients of the final multivariate latent growth model are shown in Figure 1.

## 4. Discussion

Using three-wave longitudinal data and multivariate latent growth modeling, this study examined how the home parenting environment was associated with executive function development among 5- to 6-year-old children. Three main findings emerged. First, children’s executive function increased significantly over one year, and children differed in both their initial level and rate of growth. Second, the home parenting environment showed no significant mean-level change during the study period, but there were significant between-family differences in its initial level. Third, after child sex and baseline language ability were controlled, higher initial levels of the home parenting environment were associated with both higher initial executive function and faster growth in executive function. These findings suggest that executive function development during late preschool is not only a process of average-level improvement, but also a process marked by individual variation. They further indicate that the relatively stable quality of the home parenting environment may be an important contextual correlate of such developmental differences.

### 4.1. Developmental Trajectory of Executive Function in 5- to 6-Year-Old Children

The present study found that children’s executive function increased significantly across the one-year period, with significant individual differences in both initial level and growth rate. This finding is consistent with longitudinal research showing that executive function continues to develop from late preschool into the early school years and is linked to later cognitive, academic, social, and behavioral outcomes (Reilly et al., 2022; Stucke & Doebel, 2024). Building on this literature, the present study provides evidence from Chinese 5- to 6-year-old children that executive function development during this period involves not only mean-level gains, but also variability in the pace of development. Thus, executive function in late preschool should not be viewed as a uniform maturational process; rather, children may follow different developmental patterns even within the same age period.

This result is particularly relevant in the context of the preschool-to-primary school transition. During this period, children face increasing expectations for rule-following, task persistence, sustained attention, and behavioral regulation. Growth in executive function is therefore closely related to children’s readiness for school and their engagement in classroom activities (Karimi et al., 2025; Turnbull et al., 2024). More importantly, the significant variance in the growth rate of executive function suggests that developmental change cannot be fully explained by age or general maturation alone. Some children appear to show faster gains than others, making it necessary to consider the contextual conditions that may be associated with these differences. In this regard, the observed heterogeneity in executive function trajectories provides a foundation for examining whether the home parenting environment is linked to children’s initial regulatory capacity and subsequent developmental pace.

### 4.2. Home Parenting Environment and Children’s Executive Function Trajectories

The present study found no significant mean-level change in the home parenting environment over the one-year study period, suggesting that it was relatively stable at the mean level during this time. This finding can be understood in terms of both the processes through which the home parenting environment develops and its multidimensional nature. First, core parenting behaviors, such as emotional responsiveness and language and cognitive stimulation, typically develop through sustained family interactions and are repeatedly enacted over time, thereby exhibiting considerable cross-time continuity (Dallaire & Weinraub, 2005). Second, the home parenting environment represents an integrated context encompassing multiple aspects of family life, including parenting behaviors, learning activities, the home atmosphere, and physical conditions (Gregoriadis & Evangelou, 2022; He, 2008). Even when particular parenting behaviors or family activities change over a relatively short period, changes across different dimensions may not converge into a consistent overall trend. Thus, the nonsignificant mean slope of the home parenting environment may reflect both the cross-time continuity of its core components and the aggregation of potentially divergent changes across its multiple dimensions.

Building on this trajectory pattern, the present study further examined the association between the initial level of the home parenting environment and children’s executive function development. The results showed that children with higher initial levels of the home parenting environment also had higher initial levels of executive function. This finding is consistent with the theoretical view that parenting practices such as cognitive stimulation, language input, and scaffolding jointly contribute to children’s executive function development (Hanlin & Zhou, 2024). It further suggests that differences in children’s executive function at the beginning of the study may reflect not only individual characteristics such as maturation, language ability, and sex, but also differences in the everyday family experiences accumulated before the study began.

This finding adds to existing longitudinal research by distinguishing children’s initial status from their subsequent developmental change and showing that the association between the home parenting environment and executive function was already evident in individual differences at the beginning of the study. Rather than representing only a time-specific exposure, the initial level of the home parenting environment may partly reflect children’s accumulated experiences within the family microsystem during the late preschool period. From a bio-ecological perspective, these experiences are embedded in recurring proximal processes through which children engage with caregivers in everyday family life. In higher-quality home parenting environments, children are more likely to experience responsive communication, language-rich interaction, consistent rule guidance, cognitive stimulation, and emotional support. Such experiences may provide repeated opportunities to practice attention control, goal maintenance, emotional regulation, and behavioral inhibition (Čepukienė & Janulevičė, 2025; Koşkulu-Sancar et al., 2023; Prime et al., 2023). Through repeated guidance and feedback, caregivers may help children internalize rules, maintain task goals, and regulate their responses across everyday situations. Sensitive caregiving may also support executive function by shaping developing motivational and reward-processing systems involved in self-regulation (Foley et al., 2025; Ku & Blair, 2023; Werchan et al., 2023). The observed association may therefore reflect developmental processes that unfold through sustained parent–child interactions rather than the influence of a single parenting behavior or short-term learning activity.

At the same time, the association may arise from several overlapping developmental pathways rather than from a purely direct effect of the home environment. Relatively stable family characteristics, including parental executive function and enduring patterns of parent–child interaction, may contribute to both the quality of the home parenting environment and children’s executive function (Hughes & Devine, 2019). Child-driven processes are also plausible, because children with stronger regulatory abilities may elicit more structured, responsive, and cognitively stimulating interactions from caregivers. Previous longitudinal evidence has shown that parental responsiveness can predict subsequent gains in children’s executive function, while some components of children’s executive function may also predict later changes in parental responsiveness (Merz et al., 2017). Conversely, children in less supportive or more chaotic family environments may experience fewer consistent opportunities for clear guidance and regulatory practice, which may contribute to lower executive function (Andrews et al., 2021). Thus, the findings are consistent with an accumulated proximal-process account, while shared family characteristics and reciprocal parent–child influences should also be considered plausible explanations for early individual differences in executive function.

More importantly, the initial level of the home parenting environment was also positively associated with the linear growth rate of children’s executive function. This finding moves the discussion beyond differences in initial status and highlights differences in developmental change. Whereas initial executive function may reflect children’s prior accumulated experiences, the growth rate captures variation in how quickly children’s executive function changed during the follow-up period. For 5- to 6-year-old children, the one-year study period broadly corresponds to the transition from late preschool toward primary school. During this period, children are increasingly expected to follow rules, persist in tasks, sustain attention, control behavior, wait, take turns, plan, express themselves, negotiate, and regulate emotions in everyday settings (Reilly et al., 2022).

Children in higher-quality home parenting environments may have more consistent access to clear rule guidance, appropriate opportunities for choice and autonomy, and sensitive and responsive parent–child interactions. Such experiences may provide repeated opportunities for children to practice self-regulation in daily family routines and may support the development of executive function during this transitional period (Castelo et al., 2022; Werchan et al., 2023). This interpretation is consistent with the parental socialization framework of executive function development proposed by Hanlin and Zhou (2024), which emphasizes language input, cognitive stimulation, scaffolding, and autonomy support as important family pathways for executive function development. Sensitive caregiving and positive emotional responsiveness may also provide a relational context in which children can practice emotion regulation and behavioral control with adult support (Heeman et al., 2024; Isdahl-Troye et al., 2025). Taken together, the association between the home parenting environment and the growth rate of executive function should not be understood as the immediate result of any single parenting behavior. Rather, it may reflect the role of a stable and repeatedly experienced family context in supporting children’s ongoing regulatory practice. Given the observational design of this study, these findings should be interpreted as evidence of a longitudinal association between the home parenting environment and children’s executive function trajectories, rather than as evidence of a strict causal effect.

### 4.3. Roles of Baseline Language Ability and Child Sex

The present study also examined whether baseline language ability and child sex were associated with children’s executive function trajectories. Baseline language ability, as measured by the PPVT-R, was positively associated with the initial level of executive function, but not with the linear growth rate. This finding suggests that children with stronger receptive vocabulary and language comprehension tended to show better executive function at the beginning of the study. Prior research has indicated that language ability is closely linked to executive function during the preschool years, as language comprehension may help children understand task demands, process rule information, maintain goals, regulate attention, and control behavior (Bruce et al., 2023; Filipe et al., 2023). In this sense, the association between PPVT-R scores and the initial level of executive function may reflect the role of language ability as an important individual foundation for early self-regulation.

However, baseline language ability was not significantly associated with the growth rate of executive function. This result suggests that receptive vocabulary and language comprehension may be more closely related to children’s existing level of executive function than to the pace of executive function growth over the one-year period. This interpretation is consistent with longitudinal evidence showing that the relation between language and executive function across time is not always stable and may vary depending on children’s age, assessment methods, and the specific components of executive function examined (Gooch et al., 2016; Xing et al., 2022). Therefore, individual differences in the growth rate of executive function among 5- to 6-year-old children should be understood in relation to multiple developmental conditions, including family experiences, kindergarten contexts, and maturation.

Child sex was not significantly associated with either the initial level or the growth rate of executive function. This finding indicates that, in the present study, no significant sex differences were observed in the overall mother-reported measure of executive function. Previous research on sex differences in executive function has produced mixed findings, and observed differences often depend on task characteristics, developmental stage, and measurement approach, with effect sizes generally being small (Grissom & Reyes, 2019; Silverman, 2021). Thus, the developmental differences in executive function observed in this study should not be attributed simply to child sex. Instead, the findings point to the importance of considering language-related abilities, family context, and other everyday developmental experiences when explaining variation in executive function development during late preschool.

### 4.4. Practical Implications of the Findings

The findings have practical implications for family support, kindergarten–family collaboration, and school readiness services during the preschool-to-primary school transition. First, the results suggest that children’s executive function development should not be addressed only through direct training of isolated cognitive skills. Because higher initial levels of the home parenting environment were associated with both higher initial executive function and faster growth, practitioners may need to pay greater attention to the everyday family contexts in which children practice self-regulation. Family education programs could help caregivers build stable, responsive, and cognitively stimulating home environments by encouraging consistent routines, warm parent–child communication, shared reading, guided play, appropriate autonomy support, and clear behavioral expectations.

Second, the finding that children differed in both their initial executive function and rate of growth highlights the importance of monitoring developmental change rather than relying only on one-time assessments. In kindergarten and family support settings, teachers and caregivers could work together to observe children’s attention maintenance, task persistence, emotional regulation, and behavioral control over time. Such ongoing observation may help identify children who show slower growth in self-regulation and provide them with timely support in daily routines, play-based activities, and school readiness practices.

Third, the association between baseline language ability and initial executive function suggests that language-rich interactions may be a meaningful component of early self-regulatory support. Parents and teachers can provide verbal scaffolding, explain rules clearly, ask open-ended questions, and encourage children to express their ideas during everyday activities. These practices may help children understand task demands, maintain goals, organize actions, and regulate behavior. However, because the present study used an observational longitudinal design, these implications should be interpreted cautiously. The findings do not demonstrate causal effects, but they suggest that improving the quality of children’s everyday family experiences may be a promising direction for family-based guidance and school readiness support.

### 4.5. Strengths, Limitations, and Future Directions

This study has several strengths. First, it used a three-wave longitudinal design, which made it possible to examine changes in executive function among 5- to 6-year-old children during the preschool-to-primary school transition. This design also allowed the study to distinguish between children’s initial level of executive function and their rate of growth over time. Second, the study conceptualized the home parenting environment as a multidimensional and integrated family context, rather than focusing on a single parenting behavior or isolated family factor. This approach provides a more holistic understanding of how everyday family experiences are associated with children’s executive function development. Third, the use of multivariate latent growth modeling allowed the study to examine developmental trajectories of both executive function and the home parenting environment, and to test whether the initial level of the home parenting environment was related to both the initial level and growth rate of executive function. In addition, child sex and baseline language ability were included as covariates, which helped reduce potential confounding from basic child-level differences.

Several limitations should also be noted. First, although common method bias was statistically assessed, this diagnostic procedure cannot fully address the limitations associated with reliance on a single reporter. Future studies should incorporate multiple informants and methods, including father and teacher reports, performance-based assessments of executive function, and direct observations of the home environment.

Second, the study was based on a complete-case analytic sample of children who participated in all three waves. Although attrition analyses did not indicate clear systematic bias, future research could use approaches such as full information maximum likelihood or multiple imputation to make fuller use of incomplete longitudinal data.

Third, the study followed children for only one year and primarily estimated linear change, which may have limited its ability to capture longer-term or nonlinear developmental patterns in executive function and the home parenting environment. In addition, the duration of children’s preschool education before the baseline assessment was not collected and therefore could not be included as a covariate. Future studies should extend the follow-up period, include additional measurement waves, and account for educational experience variables such as the duration of preschool education.

Fourth, the sample was drawn from one city in Jiangsu Province, China, which may limit the generalizability of the findings to children from other regions, cultural contexts, socioeconomic backgrounds, or family structures. Future studies should include more diverse samples to examine whether the observed associations vary across social and cultural contexts.

Fifth, although the longitudinal design provides evidence of temporal associations, the observational nature of the study does not allow for strong causal conclusions. Experimental, intervention, or quasi-experimental designs would be useful for testing whether changes in family support and parenting practices lead to changes in children’s executive function development.

Finally, the present study treated the home parenting environment as an integrated construct. Although this approach is useful for capturing its overall role, it may obscure the distinct contributions of specific dimensions. Future research could further examine dimensions such as emotional warmth, cognitive stimulation, rule support, neglect, interference, and punishment to clarify the specific family processes through which the home parenting environment is linked to children’s self-regulatory development.

## 5. Conclusions

This three-wave study showed that executive function among 5- to 6-year-old children increased significantly over one year, with meaningful individual differences in both initial level and rate of change. By applying multivariate latent growth modeling, the study distinguished children’s starting levels from their subsequent developmental trajectories and showed that the initial home parenting environment was associated with both the initial level and the rate of growth in executive function. This longitudinal approach extends previous research that has often examined executive function as an outcome measured at a single point in time. In addition, conceptualizing the home parenting environment as an integrated developmental context shifts attention from isolated parenting behaviors to the broader configuration of everyday family experiences, including responsive interaction, emotional support, rule guidance, learning stimulation, and activity involvement. The findings therefore suggest that executive function development during late preschool is related not only to age-related maturation but also to the wider context of children’s daily family life.

The findings also identify several practical priorities. Early childhood educators may incorporate parent guidance into regular home–preschool communication, with attention to responsive interaction, predictable routines, consistent behavioral expectations, emotion coaching, and cognitively engaging shared activities. Family support programs may translate these elements into brief and feasible guidance modules accompanied by opportunities for home-based practice. At the policy level, preschool and community services could integrate accessible parenting resources, consultation, and referral pathways into existing early childhood support systems. Future intervention studies should evaluate whether programs based on these priorities produce measurable improvements in children’s executive function. Thus, the present findings provide a basis for the development and evaluation of family-oriented support rather than direct evidence of intervention effectiveness.

## Figures and Tables

**Table 1:** Descriptive statistics and correlations among study variables.

	*M*	*SD*	1	2	3	4	5	6	7	8
1. Child sex	1.45	0.50	1.00							
2. PPVT-R	57.17	16.30	0.05	1.00						
3. EF T1	2.35	0.34	0.03	0.22 ***	1.00					
4. EF T2	2.43	0.36	0.09	0.25 ***	0.54 ***	1.00				
5. EF T3	2.49	0.36	0.09	0.29 ***	0.55 ***	0.71 ***	1.00			
6. HPE T1	3.85	0.56	0.07	0.13 *	0.29 ***	0.31 ***	0.32 ***	1.00		
7. HPE T2	3.89	0.54	0.08	0.19 **	0.32 ***	0.44 ***	0.34 ***	0.61 ***	1.00	
8. HPE T3	3.89	0.57	0.03	0.18 **	0.25 ***	0.32 ***	0.42 ***	0.58 ***	0.58 ***	1.00

Note. *M* = mean; *SD* = standard deviation; EF = executive function; HPE = home parenting environment; PPVT-R = Peabody Picture Vocabulary Test-Revised; T1 = Time 1; T2 = Time 2; T3 = Time 3. Child sex was coded as 1 = boys and 2 = girls. * *p* < 0.05. ** *p* < 0.01. *** *p* < 0.001.

**Table 2:** Fit indices for latent growth models of the home parenting environment and executive function.

Construct	Model	*χ*^2^(*df*)	*p*	CFI	TLI	RMSEA (90% CI)	SRMR
Home parenting environment	Unconditional growth model	0.85(3)	0.84	1.00	1.00	0.00 [0.00, 0.06]	0.04
Executive function	Unconditional growth model	0.53(1)	0.47	1.00	1.01	0.00 [0.00, 0.15]	0.01
Executive function	Conditional growth model	0.92(3)	0.82	1.00	1.03	0.00 [0.00, 0.06]	0.01

Note. CFI = comparative fit index; TLI = Tucker–Lewis index; RMSEA = root mean square error of approximation; CI = confidence interval; SRMR = standardized root mean square residual. In the unconditional growth model for the home parenting environment, the slope variance was fixed to zero because the freely estimated model yielded a small negative variance estimate.

**Table 3:** Path coefficients in the multivariate latent growth model.

Predictor	Outcome	*B* (*SE*)	*β*	95% CI	*p*
Home parenting environment intercept	Executive function intercept	0.304 (0.057)	0.446	[0.191, 0.417]	<0.001
Home parenting environment intercept	Executive function slope	0.082 (0.028)	0.409	[0.027, 0.138]	0.004
PPVT-R	Executive function intercept	0.003 (0.001)	0.162	[0.001, 0.005]	0.032
PPVT-R	Executive function slope	0.001 (0.001)	0.132	[−0.001, 0.002]	0.196
Child sex	Executive function intercept	0.022 (0.038)	0.043	[−0.052, 0.097]	0.554
Child sex	Executive function slope	0.013 (0.019)	0.087	[−0.024, 0.050]	0.477

Note. *B* = unstandardized coefficient; *SE* = standard error; *β* = standardized coefficient; CI = confidence interval; PPVT-R = Peabody Picture Vocabulary Test-Revised. Child sex was coded as boys = 1 and girls = 2. The intercept represents the initial level at T1, and the slope represents linear change per six-month interval.

## Data Availability

The data presented in this study are available from the corresponding author upon reasonable request due to privacy and ethical restrictions related to data from minors.

## References

[B1-behavsci-16-01415] Andrews K., Dunn J. R., Prime H., Duku E., Atkinson L., Tiwari A., Gonzalez A. (2021). Effects of household chaos and parental responsiveness on child executive functions: A novel, multi-method approach. BMC Psychology.

[B2-behavsci-16-01415] Boise C., Knoche L. L. (2024). Social–emotional competence for children with identified developmental concerns: The impact of parenting and executive function. Behavioral Sciences.

[B3-behavsci-16-01415] Bronfenbrenner U., Morris P. A., Lerner R. M., Damon W. (2006). The bioecological model of human development. Handbook of child psychology: Vol. 1. Theoretical models of human development.

[B4-behavsci-16-01415] Bruce M., Savla J., Bell M. A. (2023). From terrible twos to sassy sixes: The development of vocabulary and executive functioning across early childhood. Developmental Science.

[B5-behavsci-16-01415] Carolus A. E., McLaughlin K. A., Lengua L. J., Rowe M. L., Sheridan M. A., Zalewski M., Moran L., Romeo R. R. (2024). Conversation disruptions in early childhood predict executive functioning development: A longitudinal study. Developmental Science.

[B6-behavsci-16-01415] Castellanos-Ryan N., Parent S., Chaput-Langlois S., Rioux C., Jacques S., Simard C., Tremblay R. E., Séguin J. R., Zelazo P. D. (2023). Modelling executive function across early childhood: Longitudinal invariance, development from 3.5 to 7 years and later academic performance. Cognitive Development.

[B7-behavsci-16-01415] Castelo R. J., Meuwissen A. S., Distefano R., McClelland M. M., Galinsky E., Zelazo P. D., Carlson S. M. (2022). Parent provision of choice is a key component of autonomy support in predicting child executive function skills. Frontiers in Psychology.

[B8-behavsci-16-01415] Cheng C. H., Tein J.-Y., Shaw D. S., Wilson M. N., Lemery-Chalfant K. (2025). Predictors of stability/change in observed parenting patterns across early childhood: A latent transition approach. Early Childhood Research Quarterly.

[B9-behavsci-16-01415] Chu L., Joseph G. E. (2024). The development of executive function among monolingual English-speaking and dual language learning children in early childhood settings. International Journal of Bilingual Education and Bilingualism.

[B10-behavsci-16-01415] Cumming M. M., Poling D. V., Patwardhan I., Ozenbaugh I. C. (2022). Executive function in kindergarten and development of behavioral competence: The moderating role of positive parenting practices. Early Childhood Research Quarterly.

[B11-behavsci-16-01415] Čepukienė V., Janulevičė J. (2025). Parental discipline and self-regulation in children aged 2 to 5: A meta-analysis of research conducted from 2000 to 2022. Child & Youth Care Forum.

[B12-behavsci-16-01415] Dallaire D. H., Weinraub M. (2005). The stability of parenting behaviors over the first 6 years of life. Early Childhood Research Quarterly.

[B13-behavsci-16-01415] Diamond A. (2013). Executive functions. Annual Review of Psychology.

[B14-behavsci-16-01415] Distefano R., Nelson K. M., Palmer A. R., Masten A. S., Carlson S. M. (2024). The role of parenting in autonomy and executive function development among young children experiencing homelessness. Children and Youth Services Review.

[B15-behavsci-16-01415] Draper C. E., Yousafzai A. K., McCoy D. C., Cuartas J., Obradović J., Bhopal S., Fisher J., Jeong J., Klingberg S., Milner K., Pisani L., Roy A., Seiden J., Sudfeld C. R., Wrottesley S. V., Fink G., Nores M., Tremblay M. S., Okely A. D. (2024). The next 1000 days: Building on early investments for the health and development of young children. The Lancet.

[B16-behavsci-16-01415] Ernst J., Stelley H. (2024). Supporting young children’s self-regulation through nature-based practices in preschool. Behavioral Sciences.

[B17-behavsci-16-01415] Fan L., Qing W., Wang Y., Zhan M. (2022). Family socioeconomic status and attention deficit/hyperactivity disorder in preschool children: The mediating role of executive function. International Journal of Environmental Research and Public Health.

[B18-behavsci-16-01415] Filipe M. G., Veloso A. S., Frota S. (2023). Executive functions and language skills in preschool children: The unique contribution of verbal working memory and cognitive flexibility. Brain Sciences.

[B19-behavsci-16-01415] Foley J. E., Olino T. M., Weinraub M. (2025). On the broader significance of maternal sensitivity: Mothers’ early and later sensitive parenting matter to children’s language, executive function, academics, and self-reliance. Developmental Science.

[B20-behavsci-16-01415] Fung W. K., Chung K. K. H., Lam C. B. (2023). The longitudinal associations of household economic pressure and home chaos with children’s executive functioning, word reading, and school readiness. Child & Youth Care Forum.

[B21-behavsci-16-01415] Gioia G. A., Espy K. A., Isquith P. K. (2003). Behavior rating inventory of executive function–preschool version (BRIEF-P).

[B22-behavsci-16-01415] Gooch D., Thompson P., Nash H. M., Snowling M. J., Hulme C. (2016). The development of executive function and language skills in the early school years. Journal of Child Psychology and Psychiatry.

[B23-behavsci-16-01415] Gregoriadis A., Evangelou M. (2022). Revisiting the home learning environment: Introducing the home learning ecosystem. Australasian Journal of Early Childhood.

[B24-behavsci-16-01415] Grissom N. M., Reyes T. M. (2019). Let’s call the whole thing off: Evaluating gender and sex differences in executive function. Neuropsychopharmacology.

[B25-behavsci-16-01415] Hanlin P., Zhou Q. (2024). A unified framework of parental socialization of executive function development. Developmental Review.

[B26-behavsci-16-01415] He S.-S. (2008). Development and validation of the early childhood home parenting environment scale. Doctoral dissertation.

[B27-behavsci-16-01415] Heeman E. J., Forslund T., Frick M. A., Frick A., Jónsdóttir L. K., Brocki K. C. (2024). Predicting emotion regulation in typically developing toddlers: Insights into the joint and unique influences of various contextual predictors. International Journal of Behavioral Development.

[B28-behavsci-16-01415] Heinze H., Daseking M., Gawrilow C., Karbach J., Kerner auch Koerner J. (2025). Self-regulation in preschool: Are executive function and effortful control overlapping constructs?. Developmental Science.

[B29-behavsci-16-01415] Hughes C., Devine R. T. (2019). For better or for worse? Positive and negative parental influences on young children’s executive function. Child Development.

[B30-behavsci-16-01415] Isdahl-Troye A., Villar P., Álvarez-Voces M., Romero E. (2025). Unraveling the dynamics of emotional regulation and parental warmth across early childhood: Prediction of later behavioral problems. Scientific Reports.

[B31-behavsci-16-01415] Jiang C., Li X., Du B.-C., Huang J., Li Y., Zhang Y., Wei M., Xu X., Yang Y., Jiang H. (2024). Role of home nurturing environment on early childhood neurodevelopment: A community-based survey in Shanghai, China. BMC Pediatrics.

[B32-behavsci-16-01415] Karimi A., Poznanski B., Hart K. C., Nelson E. L. (2025). Fine motor skills, executive function, and school readiness in preschoolers with externalizing behavior problems. Behavioral Sciences.

[B33-behavsci-16-01415] Koşkulu-Sancar S., van de Weijer-Bergsma E., Mulder H., Blom E. (2023). Examining the role of parents and teachers in executive function development in early and middle childhood: A systematic review. Developmental Review.

[B34-behavsci-16-01415] Ku S., Blair C. (2023). Profiles of early family environments and the growth of executive function: Maternal sensitivity as a protective factor. Development and Psychopathology.

[B35-behavsci-16-01415] Lohndorf R., Vermeer H. J., De la Harpe C., Mesman J. (2021). Socioeconomic status, parental beliefs, and parenting practices as predictors of preschoolers’ school readiness and executive functions. Early Childhood Research Quarterly.

[B36-behavsci-16-01415] Lu T.-F., Shuai L., Zhang J.-S., Wang Y.-F., Qian Y., Zhang H.-F., Xia W.-P., Wang Z.-Y., Tan X. (2017). Reliability and validity of the Chinese version of the Behavior Rating Inventory of Executive Function–Preschool Version parent form. Chinese Mental Health Journal.

[B37-behavsci-16-01415] Memba G. V., Ostrov J. M. (2025). Executive functioning across the transition to kindergarten: Links with social and academic outcomes in early childhood. Social Development.

[B38-behavsci-16-01415] Merculief A., Tsethlikai M., Muniz F. (2024). Applying an Indigenous connectedness framework to examine environmental risk and protective factors for urban American Indian children’s executive function development. Behavioral Sciences.

[B39-behavsci-16-01415] Merz E. C., Landry S. H., Montroy J. J., Williams J. M. (2017). Bidirectional associations between parental responsiveness and executive function during early childhood. Social Development.

[B40-behavsci-16-01415] Murphy Y. E., Zhang X., Gatzke-Kopp L. (2022). Early executive and school functioning: Protective roles of home environment by income. Journal of Applied Developmental Psychology.

[B41-behavsci-16-01415] Pitta A. (2026). Executive functions in research and practice: A multimethod review of behavioral, subjective, and neurobiological assessment tools. Frontiers in Psychology.

[B42-behavsci-16-01415] Prime H., Andrews K., Markwell A., Gonzalez A., Janus M., Tricco A. C., Bennett T., Atkinson L. (2023). Positive parenting and early childhood cognition: A systematic review and meta-analysis of randomized controlled trials. Clinical Child and Family Psychology Review.

[B43-behavsci-16-01415] Reilly S. E., Downer J. T., Grimm K. J. (2022). Developmental trajectories of executive functions from preschool to kindergarten. Developmental Science.

[B44-behavsci-16-01415] Rungsattatharm L., Tasingha P., Trairatvorakul P., Chonchaiya W. (2025). Longitudinal associations between executive function and positive parenting during early childhood and resilience, self-regulation, and behavioral problems in school-age children. Child and Adolescent Psychiatry and Mental Health.

[B45-behavsci-16-01415] Sang B., Miao X.-C. (1990). Revision of the Shanghai urban trial norms for the peabody picture vocabulary test–revised. Psychological Science Communications.

[B46-behavsci-16-01415] Selman S. B., Distefano R., Dilworth-Bart J. E., Brooks-Gunn J. (2026). Child routines across preschool and associations with socioemotional adjustment. Journal of Family Psychology.

[B47-behavsci-16-01415] Silverman I. W. (2021). Gender differences in inhibitory control as assessed on simple delay tasks in early childhood: A meta-analysis. International Journal of Behavioral Development.

[B48-behavsci-16-01415] Stucke N. J., Doebel S. (2024). Early childhood executive function predicts concurrent and later social and behavioral outcomes: A review and meta-analysis. Psychological Bulletin.

[B49-behavsci-16-01415] Turnbull K. L. P., DeCoster J., Downer J. T., Williford A. P. (2024). Elucidating linkages of executive functioning to school readiness skill gains: The mediating role of behavioral engagement in the PreK classroom. Early Childhood Research Quarterly.

[B50-behavsci-16-01415] Vanhala A., Lee K., Korhonen J., Aunio P. (2023). Dimensionality of executive functions and processing speed in preschoolers. Learning and Individual Differences.

[B51-behavsci-16-01415] Venkadasalam V. P., Jenkins J. M., Ganea P. A., Wade M. (2022). Linking early maternal input during shared reading to later theory of mind through receptive language and executive function: A within- and between-family design. Journal of Experimental Child Psychology.

[B52-behavsci-16-01415] Wang S., Gai X. (2024). Bidirectional relationship between positive parenting behavior and children’s self-regulation: A three-wave longitudinal study. Behavioral Sciences.

[B53-behavsci-16-01415] Werchan D. M., Ku S., Berry D., Blair C. (2023). Sensitive caregiving and reward responsivity: A novel mechanism linking parenting and executive functions development in early childhood. Developmental Science.

[B54-behavsci-16-01415] Xing X., Wei Y., Wang M. (2022). Reciprocal relation between executive function and receptive vocabulary in Chinese preschoolers: Evidence from a two-year longitudinal study. Developmental Psychology.

[B55-behavsci-16-01415] Yang Y., Shields G. S., Zhang Y., Wu H., Chen H., Romer A. L. (2022). Child executive function and future externalizing and internalizing problems: A meta-analysis of prospective longitudinal studies. Clinical Psychology Review.

[B56-behavsci-16-01415] Zakharova M. N., Machinskaya R. I., Agris A. R. (2022). Brain executive functions and learning readiness in senior preschool age. Cultural-Historical Psychology.

[B57-behavsci-16-01415] Zhan S., Guo J. (2023). How household chaos affects social withdrawal of rural children: The indirect role of executive function and receptive language ability. Frontiers in Psychology.

